# Assessing sepsis-induced immunosuppression to predict positive blood cultures

**DOI:** 10.3389/fimmu.2024.1447523

**Published:** 2024-11-04

**Authors:** Enrique Hernández-Jiménez, Erika P. Plata-Menchaca, Damaris Berbel, Guillem López de Egea, Macarena Dastis-Arias, Laura García-Tejada, Fabrizio Sbraga, Pierre Malchair, Nadia García Muñoz, Alejandra Larrad Blasco, Eva Molina Ramírez, Xose Pérez Fernández, Joan Sabater Riera, Arnau Ulsamer

**Affiliations:** ^1^ R&D Department, Loop Diagnostics, Barcelona, Spain; ^2^ Servei de Medicina Intensiva, Hospital Universitari de Bellvitge, Institut d’Investigació Biomèdica de Bellvitge (IDIBELL), L’Hospitalet de Llobregat, Spain; ^3^ Vall d’Hebron Research Institute (VHIR), Vall d´Hebron Hospital Campus, Barcelona, Spain; ^4^ Departament de Microbiologia, Hospital Universitari de Bellvitge (IDIBELL), L’Hospitalet de Llobregat, Spain; ^5^ Research Network for Respiratory Diseases (CIBERES), Instituto de Salud Carlos III, Madrid, Spain; ^6^ Division of Emergency Laboratory, Hospital Universitari de Bellvitge, L’Hospitalet de Llobregat, Spain; ^7^ Biochemistry Core of the Clinical Laboratory, Hospital Universitari de Bellvitge, L’Hospitalet de Llobregat, Spain; ^8^ Servei de Cirurgia Cardíaca, Hospital Universitari de Bellvitge, L’Hospitalet de Llobregat, Spain; ^9^ Departament d’urgències, Hospital Universitari de Bellvitge, L’Hospitalet de Llobregat, Spain; ^10^ Banc de sang i teixits, Hospital Universitari de Bellvitge, L’Hospitalet de Llobregat, Spain

**Keywords:** bacteremia, sepsis, endotoxin tolerance, immunosuppression, blood culture, TNFα, LPS, biomarkers

## Abstract

**Introduction:**

Bacteremia is a life-threatening condition that can progress to sepsis and septic shock, leading to significant mortality in the emergency department (ED). The standard diagnostic method, blood culture, is time-consuming and prone to false positives and false negatives. Although not widely accepted, several clinical and artificial intelligence-based algorithms have been recently developed to predict bacteremia. However, these strategies require further identification of new variables to improve their diagnostic accuracy. This study proposes a novel strategy to predict positive blood cultures by assessing sepsis-induced immunosuppression status through endotoxin tolerance assessment.

**Methods:**

Optimal assay conditions have been explored and tested in sepsis-suspected patients meeting the Sepsis-3 criteria. Blood samples were collected at ED admission, and endotoxin (lipopolysaccharide, LPS) challenge was performed to evaluate the innate immune response through cytokine profiling.

**Results:**

Clinical variables, immune cell population biomarkers, and cytokine levels (tumor necrosis factor [TNFα], IL-1β, IL-6, IL-8, and IL-10) were measured. Patients with positive blood cultures exhibited significantly lower TNFα production after LPS challenge than did those with negative blood cultures. The study also included a validation cohort to confirm that the response was consistent.

**Discussion:**

The results of this study highlight the innate immune system immunosuppression state as a critical parameter for sepsis diagnosis. Notably, the present study identified a reduction in monocyte populations and specific cytokine profiles as potential predictive markers. This study showed that the LPS challenge can be used to effectively distinguish between patients with bloodstream infection leading to sepsis and those whose blood cultures are negative, providinga rapid and reliable diagnostic tool to predict positive blood cultures. The potential applicability of these findings could enhance clinical practice in terms of the accuracy and promptness of sepsis diagnosis in the ED, improving patient outcomes through timely and appropriate treatment.

## 1 Introduction

The presence of bacteria in the bloodstream is a life-threatening condition that can lead to the development of sepsis and septic shock, one of the leading causes of death in the emergency department (ED) (1–3). Bacteremia in the ED has a reported incidence of 140 to 160 cases per 100,000 person-years in the USA (4), with mortality rates ranging from 5.3% to 14.4% (5). ED patients who present with bacteremia have a higher risk of mortality at 30 days than those with negative blood cultures, which is frequently linked to delayed or inappropriate use of antibiotics (6–8).

Despite being commonly used for sepsis diagnosis, diagnostic methods such as blood cultures and biomarker tests have significant limitations. The time needed to assess the positivity of blood cultures ranges from 16 hours to a few days (9, 10), which is too long to help clinicians decide whether or not to start antibiotics. Moreover, factors such as prior antimicrobial therapy, low pathogen levels, and poor management during sampling, lead to a significant portion of false negatives (11) and false positives: Unreliable results are found in approximately 30% of samples, with large variability across facilities due to a high risk of contamination when skin or external bacteria are accidentally introduced during blood sampling (12, 13). Nevertheless, blood cultures are still the gold standard for sepsis diagnosis and the identification of possible causative organisms (14) even if its inaccuracy may lead to prolonged hospital stays and misuse of antimicrobial agents (15). Molecular tests, while faster and more sensitive, still cannot always detect clinically relevant pathogens. Therefore, ED practitioners need new tools to identify patients with bacteremia causing sepsis accurately, and as quickly as possible.

There are some point-of-care biomarker tests available that can be easily performed directly from blood samples, most of which are based on the identification of serum biomarkers such as Procalcitonin (PCT), C-reactive protein (CRP), or Pancreatic Stone Protein (PSP). The main disadvantage of these biomarkers is their low specificity, as positive results can be induced by other noninfectious conditions (16–18). Other molecular techniques, such as rapid microbiological panels, have a higher sensitivity than blood cultures. However, the higher performance of T2Bacteria (T2 Biosystems, c., USA) (19), a rapid molecular test for bacteria, cannot help clinicians detect the main immunological derangements that define the presence of sepsis caused by those pathogens.

Consequently, bacteremia prediction models have evolved. The first models rely on the presence of fever and high levels of inflammatory markers, such as CRP and neutrophil count (4, 20, 21). These variables are not sufficiently accurate to predict bacteremia. Shapiro’s predictive algorithm have been used in some settings (22), though current evidence does not support its clinical validity used as a single tool to rule in or rule out the diagnosis of sepsis. In recent years, new algorithms based on electronic health records (EHRs) and machine learning have increased the number of variables included and the accuracy of sepsis diagnosis, although they are not widely applicable in clinical practice (23–25). These algorithms use data from baseline clinical characteristics and laboratory results.

During sepsis, it is described that patients develop an immunosuppression phase, also called endotoxin tolerance (ET), whose physiological purpose is to prevent overwhelming inflammation caused by the presence of pathogens in the blood, but also having potentially detrimental effects such as failure to eradicate the primary infection or facilitating secondary ones (26–28). In healthy patients, LPS activates toll-like receptor (TLR) family members, (in particular, TLR4 is the most broadly described) leading to the activation of NF-κB, MyD88, IRF3, and the production of pro-inflammatory cytokines (29, 30). During endotoxin tolerance, immune cells exhibit a transient state in which they cannot adequately respond to endotoxin challenges through this pathway (29). Also *in vitro* studies have demonstrated that immune cells from patients with endotoxin tolerance are unable to trigger an adequate innate immune response when challenged with endotoxin (31).

The study of the activation of the innate immune system by *in vitro* stimulation of whole blood with TLR-specific agonists could improve the performance of machine learning models and other sepsis prediction algorithms. However, the proportion of ED patients with bacteremia who present with immunosuppression has not been properly characterized, even though its study has the potential to contribute to an early detection of sepsis.

We aimed to measure specific biomarkers related to innate immune activation in response to a LPS stimulus as a new tool to characterize sepsis patients according to their innate immune system activation status and to predict positive blood cultures.

## 2 Materials and methods

### 2.1 Patient recruitment

Sepsis-suspected patients who fulfilled the diagnostic criteria for Sepsis-3 of the Society of Critical Care Medicine and the European Society of Intensive Care Medicine international conferences (32) were included (both cohorts). The criteria for preinclusion assessment were adult patients aged 18 years or older presenting signs of systemic infection on admission to the ED. The SOFA score and the classification of sepsis according to the Sepsis-3 criteria were determined within the first 24 hours following patient admission. Blood samples were collected at the time of ED presentation and before any treatment was administered. Exclusion criteria encompassed patients with active chronic inflammatory diseases, including autoimmune diseases or chronic inflammatory conditions (except controlled asthma). Patients with hematological malignancies or active cancers under treatment were excluded, as were those with immunosuppression due to prior or current treatment with systemic steroids and/or immunosuppressive drugs within the last month. Severe hepatic dysfunction, evidenced by advanced liver disease (e.g., cirrhosis with Child-Pugh C classification), and severe renal dysfunction, such as end-stage renal disease or patients on dialysis, were also grounds for exclusion. Additionally, patients with chronic viral infections—including a known diagnosis of HIV/AIDS or active hepatitis B or C infections—were excluded, as were pregnant or lactating women. Patients with organ transplants, intravenous drug use, or any condition that, in the investigator’s judgment, could compromise safe participation in the study were also not included. The study was approved by the Ethics Committee of Bellvitge University Hospital (PR358/20).

The baseline clinical characteristics for each group of the reference cohort are described in **Supplementary Table 1**. Signed consent was waived by the institutional ethics committee, as only anonymized data were used for this part of the study. Patients who presented with bacteremia were significantly older than those with negative blood cultures (p value < 0.05). Validation cohort was enrolled after the reference cohort was completed and included ED patients whose blood cultures were positive (**Supplementary Table 2**). In this case, blood collected at the time of presentation to the ED (stored less than 24 hours) was used for LPS (10 ng/ml) challenge and TNF-α measurement. Blood samples were obtained from anonymous healthy donors and asymptomatic patients scheduled to undergo elective cardiac surgery requiring cardiopulmonary bypass as controls for the validation cohort. These patients agreed to participate in the study, taking advantage of the remaining blood after extraction of the routine or scheduled laboratory analysis on admission.

### 2.2 Cytometry

To detect and characterize monocytes, anti-CD14, fluorescein isothiocyanate–conjugated α human leukocyte antigen–antigen D–related (anti-HLA-DR), and anti-CD3 (Immunostep^©^, Salamaca, Spain) antibodies were used [see **Supplementary Figure 4** and (33)]. The samples were analyzed with a FACSCanto flow cytometer (BD Biosciences) and FlowJo vX. 0.7 software (FlowJo^©^, LLC, BD, New Jersey, U.S.A.). Tumor necrosis factor–α (TNFα), interleukin (IL)–1β, IL-6, IL-8, and IL-10 protein levels were determined using a human inflammatory cytometric bead array (CBA) kit (BD Biosciences^©^, New Jersey, U.S.A.) following the manufacturer’s protocol. The samples were collected by flow cytometry using a BD FACSCanto flow cytometer (BD Biosciences^©^, New Jersey, U.S.A.). WBC, lymphocyte, monocyte, and neutrophil total count and percentage were obtained by a FACSCanto flow cytometer (BD Biosciences^©^, New Jersey, U.S.A.), and FlowJo vX.0.7.

### 2.3 Reagents

The TLR-specific agonists x-1 and x-2 (both TLR2/TLR6-specific) are purified preparations of lipoarabinomannan (LTA)-like components; K and I (TLR2-TLR4-specific) agonists are purified preparations of lipopolysaccharides (LPS) and peptidoglycan (PGN)-like components. Lipopolysaccharide (LPS) (TLR4 specific) from *Salmonella abortus* and *Escherichia coli* were obtained from Sigma. Roswell Park Memorial Institute (RPMI) medium (Invitrogen) was used to dilute the whole blood samples 1:1. After ex vivo TLR agonist stimulation, whole-blood samples were stored at -80°C and quantified by cytometric bead array.

### 2.4 Statistical analysis

The normality of the distribution of quantitative variables was assessed using the Kolmogorov-Smirnov test. Comparisons of quantitative variables between two groups were performed using the Mann-Whitney U test for non-normal distributions and the unpaired two-tailed Student’s t-test for independent samples in cases of normal distribution. Data were analyzed using SPSS statistical software version 25.0 (IBM Corp., Armonk, NY, USA).

## 3 Results

### 3.1 Evaluating various TLR stimulators to establish endotoxin challenge test conditions

#### 3.1.1 Evaluation of different TLR stimulators

TNFα is induced upon LPS stimulation in whole blood obtained from healthy individuals (34, 35). However, other pathogen-associated molecular patterns (PAMPs), in addition to LPS, are recognized by toll-like receptor-4 (TLR4) and might be good candidates for TLR stimulators (36). To determine the optimal conditions for ET detection, we aimed to identify which TLR stimulators were most effective at eliciting innate immune responses. Blood samples from 5 healthy donors were stimulated with different Toll-Like Receptor (TLR)-specific agonists, namely, TLR4-specific agonists; x-1 and x-2, TLR2-TLR6-specific agonists; and K and I, TLR2-TLR4-specific agonists, and were compared to LPS, which is the PAMP most commonly used as an effector. The cells were incubated for 3 hours, after which the TNFα levels were analyzed. As shown in **Supplementary Figure 1A**, LPS induced the production of significant levels of TNFα, while the non-TLR4 agonists x-1 and x-2 barely induced the production of the cytokine. The TLR2-TLR4-specific agonists K and I did not significantly increase the production of TNFα, even at concentrations higher than those of LPS. This demonstrates that only TLR4-specific agonists are useful for characterizing the innate immune response, and among them, LPS has the greatest potential.

#### 3.1.2 Comparison of two different bacterial LPS sources

Another point to be considered to optimize the conditions for challenging the innate immune system is identifying the best LPS source for the procedure. Therefore, the ability of LPS from *E. coli* and S. *abortus* to induce a cytokine response was compared. TNFα production after ex vivo *E. coli* and *S. abortus* LPS stimulation (10 ng/mL, 3 h) of hospitalized non-septic patients’ whole blood was quantified by cytometric bead array (n=25). As shown in **Supplementary Figure 1B**, no differences were detected when blood samples were stimulated with different LPS compounds. Therefore, *S. abortus* LPS (10 ng/mL) was selected for further stimulation experiments due to its greater availability.

### 3.2 Variable identification in the reference cohort

Once optimal endotoxin challenge conditions were defined, blood samples were obtained from 41 patients who fulfilled the Sepsis-3 criteria at ED admission. Blood was sent straight to the hospital facilities for analysis, except for one 1 ml aliquot that was challenged with LPS (see methods). After obtaining the blood culture results, 22 patients were negative, and 19 were positive for bacteremia.

The baseline variables most used for predicting bacteremia were collected. Temperature, heart rate, respiratory rate, pH, bicarbonate levels, S_a_O2, Lactate, Acute Physiology and Chronic Health Disease Classification System II (APACHE II) score, Quick-SOFA score, and Glasgow score were obtained from the hospital EHR. Notably, S_a_O2 was significantly reduced in patients with bacteremia, and the APACHE II score, Quick-SOFA score, and Glasgow score were significantly increased. Serum CRP and PCT were measured by standard biochemistry, and no significant differences were detected between the groups. White blood cell, lymphocyte, monocyte, and neutrophil total counts and percentages were also measured. Interestingly, only the monocyte population was reduced in the bacteremia cohort. Basal HLA-DR was also measured, and no differences were found between the groups (**Table 1**).

**Table 1:** Reference cohort basal variables.

Variable	Bacteremia negative (n=22)			Bacteremia positive (n=19)			p.value
	Mean (SD)	Median [Q1-Q3]	*missing*	Mean (SD)	Median [Q1-Q3]	*missing*	
Temperature	37.81 (1.44)	38.4 [37.78-38.5]	0	37.73 (1.59)	38.1 [36.4-38,6]	0	*0.674*
Heart rate	99.5 (20.91)	105 [84.25-110.75]	0	106.5 (25.22)	110 [88-124.5]	0	0.339
Resp. rate	24.14 (5.55)	22 [20-29.50]	0	25.47 (5.45)	26 [20-30]	0	0.443
SaO2	94.82 (4.26)	96 [93.25-98]	0	91.84 (5.05)	92 [90-95]	0	**0.047**
APACHE II	14 (5.12)	14 [11.25-16]	0	17.84 (5.55)	17 [14-22.5]	0	**0.027**
Q-SOFA	1.41 (0.73)	2 [1-2]	0	1.95 (0.85)	1.5 [2-2.5]	0	**0.035**
Glasgow	14.41 (1.18)	15 [14-15]	0	12.84 (1.98)	13 [11.5-15]	0	**0.003**
pH	7.42 (0.07)	7.42 [7.37-7.46]	0	7.4 (0.07)	7.41 [7.36-7.45]	0	0.466
HCO3-	24.36 (4.63)	23.3 [21.55-26.83]	0	22.18 (4.06)	21 [19.05-25.2]	0	0.121
Lactate	2.98 (1.44)	2.7 [2.03-4.05]	0	3.66 (1.99)	3.4 [2-4.8]	0	0.213
CRP	164 (115)	163 [70-248]	0	176 (98)	180 [107-270]	0	0.708
PCT	10.06 (20.19)	1.74 [0.23-4.5]	0	14.23 (17.31)	7.73 [2.24-16.54]	4	*0.061*
WBC	17810 (7504)	17450 [12750-22625]	0	14959 (8631)	15400 [8750-18700]	0	0.265
Lymphocyte	1221 (932)	920 [575-1332]	0	914 (583)	830 [500-1055]	0	0.222
Monocyte	1111 (849)	950 [593-1268]	0	519 (313)	570 [330-685]	0	**0.007**
Neutrophil	15477 (7231)	15150 [10098-19850]	0	14017 (7841)	12800 [9450-17100]	0	0.112
HLA-DR (Basal)	163.1 (147.8)	110.5 [73-224]	2	111.01 (74.57)	94.9 [625-150]	4	*0.333*

Temperature is expressed in Celsius degrees (°C). Heart rate is expressed in beats per minute, Respiratory rate is expressed in breaths per minute, SaO_2_ (functional oxygen saturation) is expressed in %, APACHE II=Acute Physiology and Chronic Health disease Classification System II, Q-SOFA = quick Sepsis Related Organ Failure Assessment, Glasgow states for Glasgow coma score, HCO_3_^-^ (Bicarbonate) is expressed in mmol/L, Lactate is expressed in mmol/L, CRP (C-reactive protein) is expressed in mg/L, PCT (Procalcitonin) is expressed in µg/L. WBC (white blood cell count), Lymphocyte, Monocyte and Neutrophil are expressed in cells per microliter. HLA-DR values are cell-bound HLA-DR on monocytes expressed as mean intensity fluorescence (MIF) in arbitrary units (A.U.). Values in italics follow a non-normal distribution and have been analyzed by Mann-Whitney U Test. Values in bold have a p.value <0.05.

**Figure 1:**
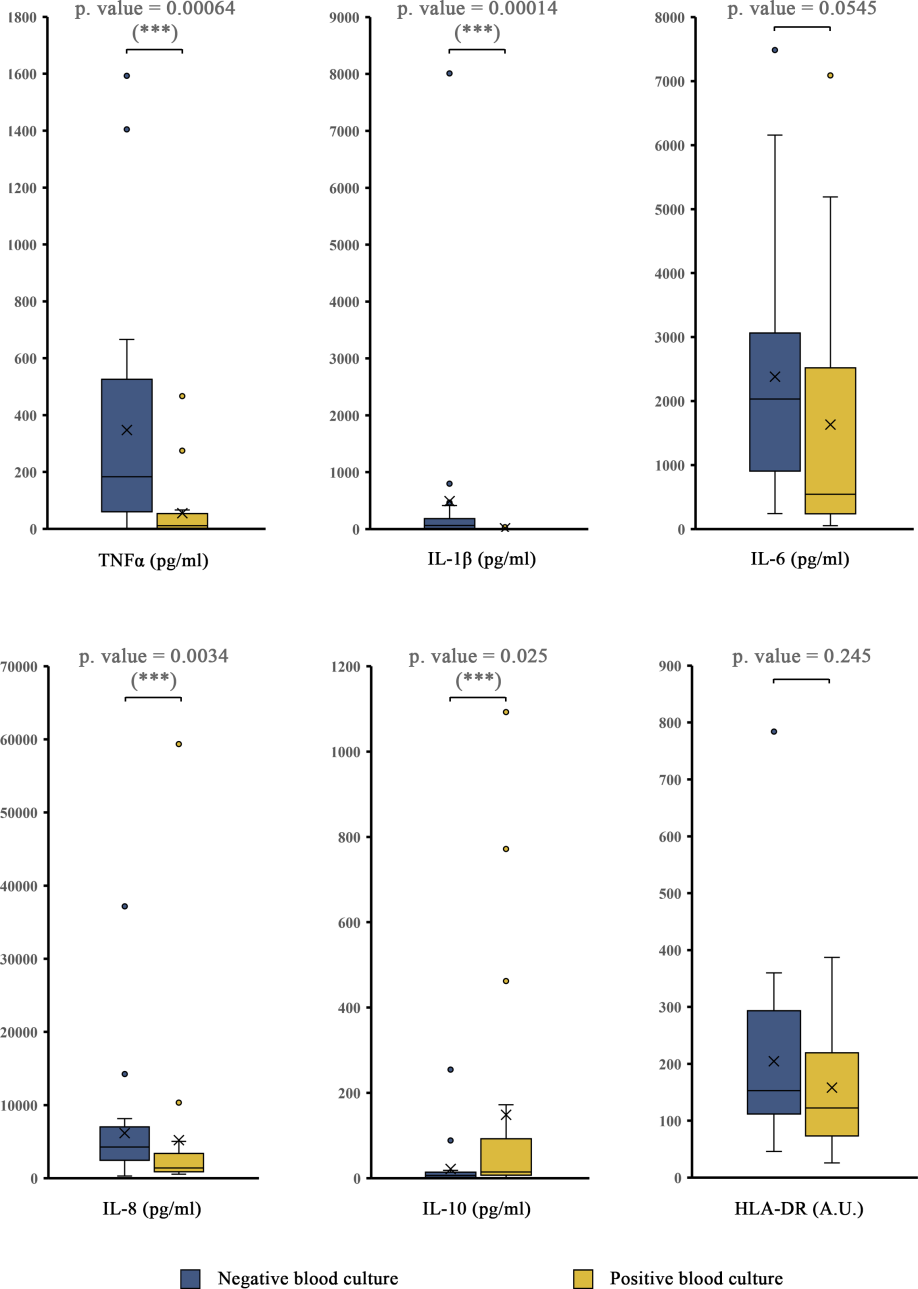
Quantification of biomarkers in the reference cohort after LPS challenge. TNFα, IL-1β, IL-6, IL-8, IL-10, and HLA-DR whole-blood production after *ex vivo B*. *abortus* LPS stimulation (10 ng/mL, 3 h) was quantified by cytometric bead array, comparing patients whose blood culture results were positive to patients whose blood culture results were negative. Box plots showing the medians, interquartile ranges (IQRs), minimums and maximums by group. Values over the maximum +1.5x IQR or below the minimum -1.5xIQR are considered outliers and are plotted outside the minimum and maximum range. X indicates the mean. *** = p<0.05; according to the Mann-Whitney U test. HLA-DR values are cell-bound HLA-DR on monocytes expressed as mean intensity fluorescence (MIF) in arbitrary units (A. U.).

Subsequently, potential new variables of interest upon whole blood LPS challenge were identified. A sample of 1 ml of whole blood obtained at ED admission was challenged *ex vivo* with LPS (10 ng/mL) for 3 h, and the production of TNFα, IL-1β, IL-6, IL-8, and IL-10 was quantified by cytometric bead array. A significant decrease in TNFα, IL-1β, and IL-8 production was observed in patients with documented bacteremia (p value < 0.05), and a significant increase in IL-10 was also detected (p value < 0.05). HLA-DR levels were measured and compared to basal values, although no significant differences induced by endotoxin exposure were found (**Figure 1**).

Some previous works defined a TNFα cut-off value of 200 pg/ml upon LPS challenge to define ET (37, 38). Although this threshold has been stablished in almost exclusively pediatric population and is not yet widely used, we performed statistical measures of sensitivity, specificity and NPV (Negative Predicting Value), by using this cut-off value (see **Supplementary Figure 2**). Moreover, ROC curve was plotted and AUC (Area Under the Curve) was estimated (see **Supplementary Figure 3**).

### 3.3 Validation cohort

To confirm low TNFα production after LPS challenge as a feature of bacteremia, we measured this response in a new ED cohort of patients whose blood cultures were positive within the first 24 hours of ED admission (n = 35). The average age of this cohort was 74.3 years, and 40% were female (**Supplementary Table 2**). Anonymous healthy volunteer (HV) donors (n = 10) and non-septic patients scheduled for cardiopulmonary bypass surgery (CS, n = 48) were enrolled as controls. The cardiac surgery group was selected due to the routine performance of preoperative assessments to corroborate an otherwise healthy condition, fitness for elective surgery, and the absence of sepsis. The average age of this group was 66.5 years, and 20.5% were female.

**Figure 2:**
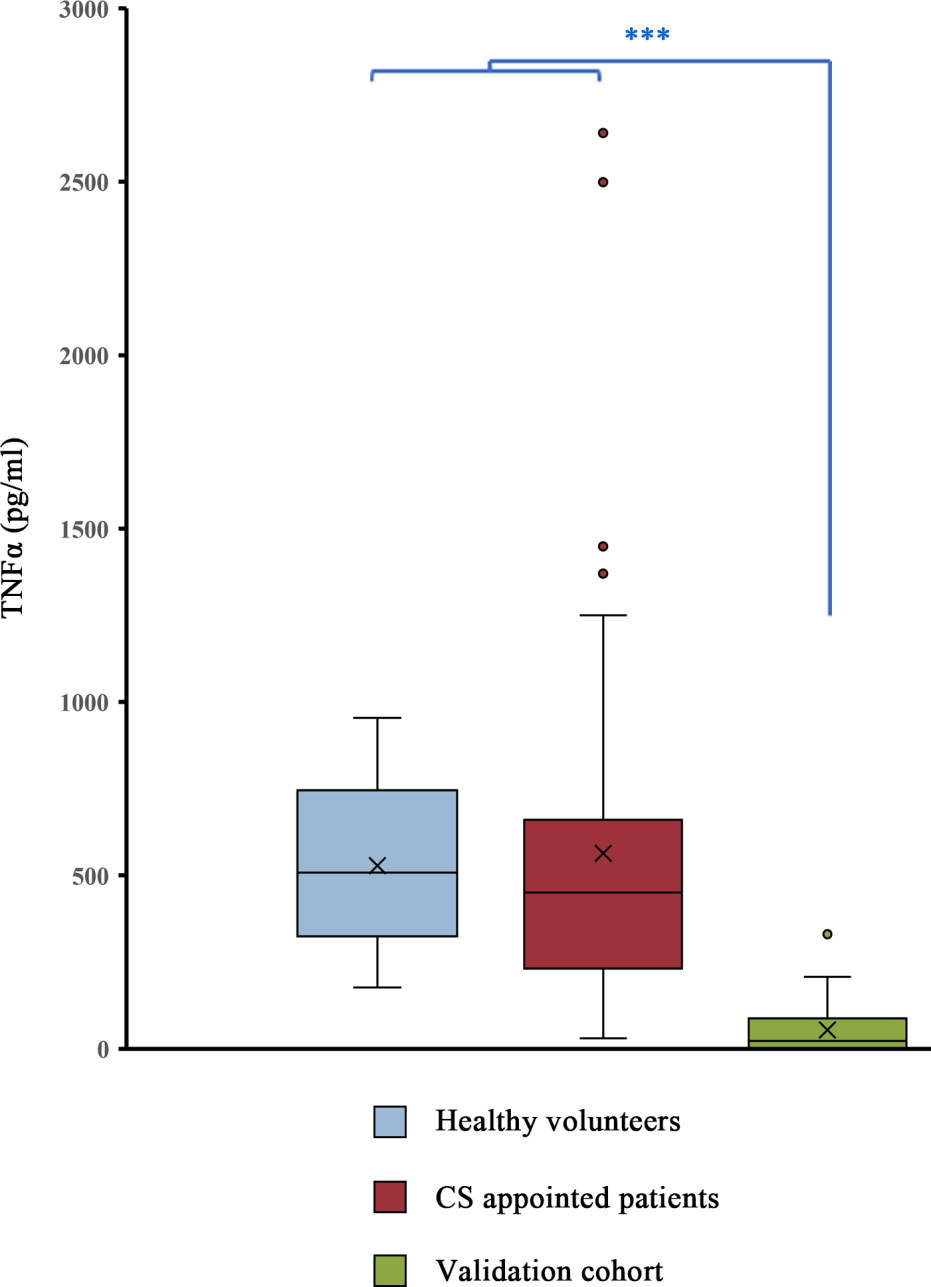
The ED patients in the validation cohort (green) were compared with healthy volunteers (anonymous donors) and patients who were scheduled to receive cardiac surgery (CS) the next day. Quantification of TNFα after LPS challenge (10 ng/mL, 3 h) by cytometric bead array. Box plots showing the medians, interquartile ranges (IQRs), minimums and maximums by group. Values over the maximum +1.5x IQR or below the minimum -1.5xIQR are considered outliers and are plotted outside the minimum and maximum range. X indicates the mean. *** = p<0.05 according to the Mann-Whitney U test.

**Figure 3:**
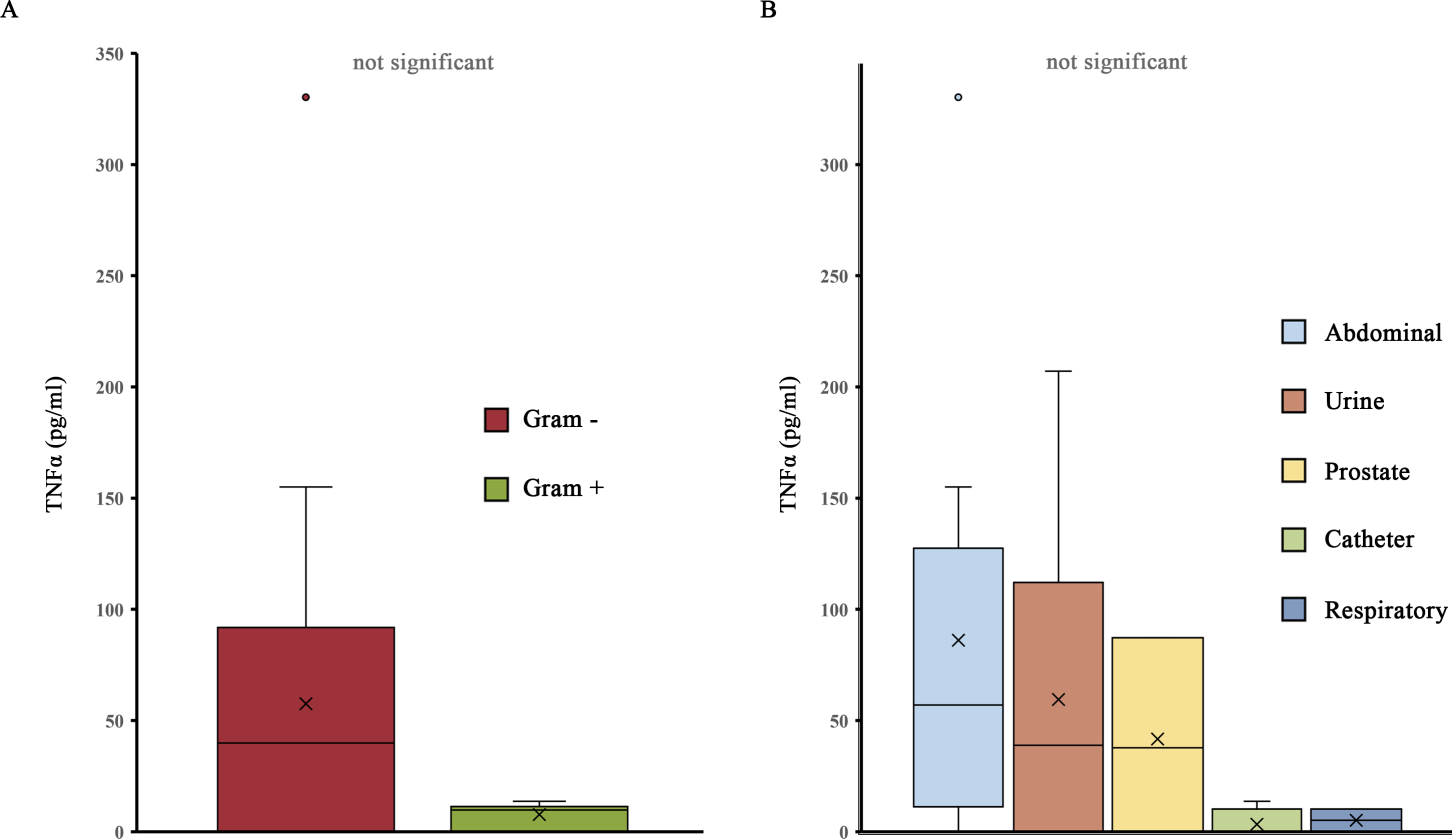
The ED patients in the validation cohort were grouped by feature. **(A)** Grouped according to Gram+ or Gram– infection status (excluding 2 patients who had a mixture of both). Quantification of TNFα after LPS challenge (10 ng/mL, 3 h) by cytometric bead array. Box plots showing the medians, interquartile ranges (IQRs), minimums and maximums by group. Values over the maximum +1.5x IQR or below the minimum -1.5xIQR are considered outliers and are plotted outside the minimum and maximum range. X indicates the mean. P values were obtained via unpaired, two-tailed t tests. **(B)** Groups according to the suspected primary infection site. Quantification of TNFα after LPS challenge (10 ng/mL, 3 h) by cytometric bead array. Box plots showing the medians, interquartile ranges (IQRs), minimums and maximums by group. Values over the maximum +1.5x IQR or below the minimum -1.5xIQR are considered outliers and are plotted outside the minimum and maximum range. X indicates the mean.

There was significantly lower TNFα production after LPS challenge in patients with bacteremia than in HV (p = 1.1 × 10^-6^) or CS patients (p = 5.14 × 10^-11^) (**Figure 2**). While patients with bacteremia produced an average of 54.1 (+/-72.5) pg/ml, HV and CS patients produced an average of 528.3 (+/-258.7) pg/ml and 563.8 (+/-565.1) pg/ml, respectively. In 26 patients, only gram-negative bacteria were detected; in 7 patients, only gram-positive bacteria were detected; and in 2 patients, both gram-negative and gram-positive pathogens were detected. Comparing gram-negative vs gram-positive patients, no significant difference in ET was observed in patients with sepsis caused by either type of bacteria (p value = 0.122) (**Figure 3A**). No significant differences were detected between the different suspected primary sources (**Figure 3B**).

## 4 Discussion

This study reports a significantly lower TNFα production after LPS challenge in patients with sepsis and bacteremia than in healthy volunteers and patients undergoing elective cardiac surgery. We showed that ET, which reflects the immunosuppressive state of the innate immune system (26, 34), can contribute to the early diagnosis and characterization of patients with bacteremia and sepsis. Failure to respond to an LPS challenge is correlated with sepsis and can predict a worsening condition if prolonged over time (39).

The success of sepsis management depends on prompt and accurate interventions. Although scientific progress has been made in recent years, a widely accepted method for the early prediction of positive blood cultures in clinical practice is lacking. Identification of new parameters to define the presence of bacteremia in patients is an urgent priority. Baseline variables, including vital signs, are widely used to predict bacteremia, although they are not specific and can be influenced by ongoing treatments. For instance, arterial blood pressure and heart rate may be affected by vasopressors or antihypertensive drugs, and oxygen delivery may affect blood oxygen saturation and respiratory rate. Models based on complete blood count (CBC) and differential count test (CBC/DC test) showed promising results in predicting positive blood cultures, particularly when combined with other parameters such as CRP (40). Similar results were obtained in neonates (41).

The availability of algorithms that include a combination of clinical and laboratory variables to reliably predict the presence of bacteremia in sepsis patients is a cornerstone in emergency medicine. A few years ago, an algorithm was used to predict bacteremia based on the EHR data of 41280 adult patients, and this algorithm has been further validated in 4 different cohorts (24, 25). The main predictor variables are clinical parameters, such as temperature, creatinine, and CRP, including a total of 49 features in its simplified version. This model achieved AUROCs ranging from 0.75 to 0.81 in different cohorts. Other models based on machine learning have been developed, but only a few have been validated (43).

There have been other attempts to identify new variables that can contribute to improving the current algorithms. A model developed through machine learning obtained remarkable results in predicting positive blood cultures by including CBC/DC tests together with cell population data (CPD) through flow cytometric parameters such as side scatter light (SSC) and forward scatter light (FSC), allowing features such as cellular volume, granularity, density, or membrane surface to be obtained (43, 44).

Most positive blood culture prediction models have been developed in the ICU setting, reaching a much higher prediction rate (45, 46). Only a few algorithms have been designed and validated for ED patients, where heterogeneity is even higher (20, 25). Our results may improve the current algorithms, as it is highly unlikely that a patient who responds normally to LPS challenge would have a positive blood culture.

The challenge of immune cells with endotoxin allows the gathering of information on the immune state of patients that cannot be obtained otherwise. We investigated the role of several molecules involved in the ET stage after LPS challenge of whole blood and identified TNFα as a promising biomarker and a new variable to be included in modern algorithms for sepsis classification. TNFα after ET challenge shows similar sensitivity for gram-positive and gram-negative bacteria. The combination of TNFα with other cytokines, such as IL-1β, IL-8, or IL-10, did not increase sensitivity. Yet, the significant variation in IL-1β, IL-8, and IL-10 levels upon LPS challenge make them good candidates for further exploration in future works.

Some reports have identified immunoparalyzed patients by detecting a reduction in HLA-DR expression in circulating monocytes and observing a consistent reduction in HLA-DR in monocytes in sepsis patients (47, 48). According to Leventogiannis et al. (49), a reduction in HLA-DR receptor/monocyte ratio of less than 50000 can occur in approximately 42% of patients with different types of infection who fulfill the sepsis-3 criteria. Interestingly, a reduced expression of HLA-DR in monocytes correlates with an impaired capacity to present antigens effectively (50), indicating its potential role in measuring adaptative immunity activity rather than in innate immunity. This finding aligns with our observations that monocyte HLA-DR was not significantly reduced at baseline or after LPS stimulation in samples from ED patients whose blood cultures were positive. A correlation between ET and reduced monocyte HLA-DR expression in sepsis patients has been described in several studies (51, 52). LPS challenge and monocyte HLA-DR expression showed potential in predicting the risk of nosocomial infections in pediatric patients (37). These two biomarkers are similarly reduced in critically ill patients with sepsis or after trauma or surgery (39). Notably, persistence in this reduction is a predictor of more severe outcomes.

The production of several cytokines is impaired upon LPS challenge in sepsis patients with bacteremia. The dynamics of IL-10 are opposite to those of TNFα, although this variation is less sensitive. The correlation between IL-6 and TNFα production after LPS challenge was previously described (53). A decrease in TNFα production after LPS challenge is a good indicator of bacteremia, as a decrease in TNFα production and in the number of circulating monocytes has been reported to correlate with bacteremia (54).

The results of this study are relevant to continue the study of the clinical applicability of ET test and further change clinical practice on early diagnosis of sepsis. Sepsis-3 criteria are not sensitive or specific enough to confirm or rule out bacteremia causing sepsis at presentation of patients to the ED. Overt clinical signs of organ dysfunction are extremely late markers of an underlying complicated infectious process leading to dead. To date, there are zero objective tools quickly available in clinical practice to confirm or refute a suspicion of sepsis at time of presentation to the ED. Clinical data, clinicians’ criteria and other late markers of organ dysfunction (lactate, creatinine, hypotension) or unspecific inflammation markers (leucocytes, CRP, etc.) are not useful to triage ED patients with a potential infection leading to sepsis. Blood cultures are usually too slow for the early diagnosis of sepsis, although, when eventually positive, can help target antimicrobial treatment when patient´s clinical condition improves. Rapid molecular tests could detect infections or bacteremia, though it does not imply the presence of sepsis and their false-positive rates leading to overdiagnosis are concerning. Improving triage of patients with an infection who are incubating a bacteremia and sepsis (detection of ET pre-clinically or in patients with established organ dysfunction) in the ED, will allow clinicians to intervene priority patients with a positive test to suppress the development of further events leading to clinical deterioration and organ dysfunction. The early diagnosis of high-risk patients, detecting sepsis-induced immunosuppression state with ET would substitute the current late clinical and laboratory diagnosis of sepsis, potentially improving patient outcomes.

The identification of culture negative sepsis is a clinically relevant finding of our work. In some patients of this study, ET test was positive in some patients fulfilling sepsis-3 and SOFA criteria who later had negative blood cultures. In clinical practice this situation occurs in up to 60% of sepsis cases (55), and the diagnosis can be overlooked and detected later when overt clinical signs of sepsis or septic shock are present. Despite innate immune system suppression has been observed in other inflammatory conditions, such as severe trauma, major surgery, burns, viral infections, and pancreatitis (39, 56–59), a significant and prolonged failure to produce TNFα upon LPS challenge is a specific parameter of bacteremia and sepsis, which is certainly an advantage of this assay. This potential application needs further study for validation.

Patients fulfilling the sepsis-3 criteria and whose blood culture is negative, can have an underlying serious condition. We cannot provide sufficient evidence to suggest that a negative ET could skip broad spectrum antibiotics in patients who meet sepsis-3 criteria. Moreover, further validations in clinical studies are needed to have a clear framework for the use of ET test in these patients, as the delay in empiric antibiotic administration leads to increase mortality in sepsis (60, 61).

The main relevance of ET test relies on its potential to be easily and quicky implemented as a point-of-care test for the early triage of patients with infections presenting to the ED, and to rule out sepsis in those patients who have an overwhelming inflammatory response not due to sepsis, and therefore, to consider non-complicated infections or other alternative diagnoses when the test is negative. Rapid microbiological tests could be complimentary to ET test to delineate the etiology of the immunological diagnosis of sepsis, and to further narrow antimicrobial treatment. The development of point-of-care devices for the detection of ET specific for sepsis at time of presentation to ED triage and used together with novel rapid microbiological tests is promising. It could improve current algorithms or be combined with other tools, such as MALDI-TOF or other molecular diagnostics.

Also, the detection of immunoparalysis may have potential for application in predictive enrichment and personalized therapeutic approaches to anticipate clinical responses to immunostimulatory therapy (62). For instance, interferon γ (IFNγ) has been proposed as a potential therapeutic approach to reverse immunoparalysis, although the best strategy for monitoring its effects is unknown (49, 63, 64). Other potential drugs that could be used to reverse immunoparalysis include GM-CSF (26), IL-4 (65), IL-7 (66), IL-15 (67) or immunoglobulin (68). However, none of them led to a clear clinical benefit so far (42).

Our work has several limitations. First, as in other studies (24), we did not consider other medications the patients were receiving at the time of the study beyond those specified in the exclusion criteria. Additionally, our validation cohort included healthy patients. In subsequent studies, we plan to select patients who fulfill the sepsis-3 criteria and correlate the presence of ET between bacteremia-positive patients and bacteremia-negative patients. Second, the onset of sepsis timing and the duration of innate immune system paralysis are unknown, leading to suboptimal characterization of different subsets of sepsis patients in this report, however, this was not the main objective of the study at this phase. Finally, we acknowledge that the small sample sizes in both cohorts limit our ability to thoroughly characterize responses across different patient subgroups. Therefore, further studies with larger and more diverse patient populations are essential to validate and generalize the findings presented here.

In conclusion, challenging whole blood cells with LPS allows the identification of a hitherto largely overlooked variable, such as ET, as a variable of interest for the early diagnosis of bacteremia and sepsis. This feature is a good candidate for inclusion in further algorithms aimed at timely prediction of positive/negative blood cultures in ED patients. New tools are needed to predict sepsis and bacteremia accurately and as quickly as possible in the ED. Further clinical studies on sepsis-induced immunosuppression at the bedside are urgently needed.

## Data Availability

The raw data supporting the conclusions of this article will be made available by the authors, without undue reservation.
