# Supplementary material for: Assessing sepsis-induced immunosuppression to predict positive blood cultures
Source: Front Immunol. 2024 Nov 4;15:1447523. doi: 10.3389/fimmu.2024.1447523 (PMC11570276; doi:10.3389/fimmu.2024.1447523)
Supplement: Supplementary file 1 [file DataSheet1.pdf]

Supplementary Figure 1:

A)

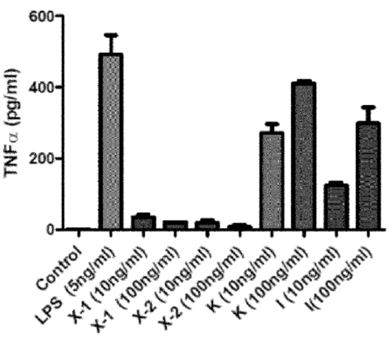

B)

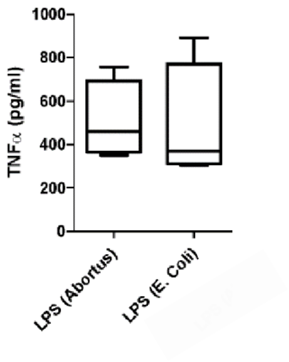

Suppl. figure 1 A) TNFα production in whole blood obtained from 5 healthy donors upon exposure to different stimulators. TNFα (pg/ml) was measured after 3 hours of *ex-vivo* stimulation: [LPS, 5ng/ml, TLR4 specific]; [x-1 and x-2, 10 and 100ng/ml, TLR2-TLR6 specific]; [K and I, 10 and 100ng/ml, TLR2-TLR4 specific] B) Quantification of TNFα production in whole blood from 25 healthy patients after exposure to 10 ng/ml LPS obtained from either E. coli or B. abortus. TNFα (pg/ml) was quantified by cytometric bead array. No significant differences between different LPS sources were observed.

Supplementary Figure 2:

	Blood culture	
	Negative	Positive
ET	11	17
No ET	11	2

Sensitivity = 89.47% [66.86% to 98.70%]

Specificity = 50% [28.22% to 71.78%]

Negative Predictive Value = 84.62% [58.15% to 95.61%]

Suppl. figure 2. A cut-off of TNFα 200 pg/ml upon LPS challenge has been stablished in the literature -especially in pediatric patients- to define immunoparalysis (Hall et al, 2010; Cornell et al, 2012). Here we challenged the reference cohort results considering TNFα below 200 pg/ml upon 10 ng/ml LPS challenge as cut-off value for Endotoxin Tolerance (ET). [95% Confidence Interval]

Hall 2010: DOI: 10.1007/s00134-010-2088-x

Cornell 2012: DOI: 10.1016/j.jtcvs.2011.09.011

Supplementary Figure 3:

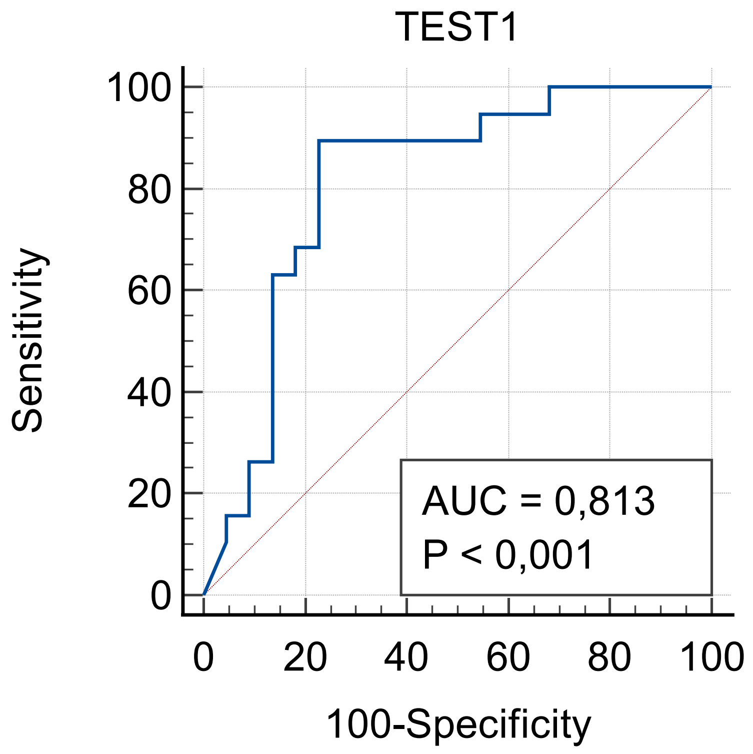

AUC = 0.813 [0.661 to 0.918]

Suppl. figure 3. ROC (receiver operating characteristic) Curve and AUC (Area Under the Curve) [95% Confidence Interval] on reference cohort results (positive vs negative blood culture) by DeLong methodology (DeLong et al., 1988)

https://www.medcalc.org/manual/roc-curves.php

DeLong ER, DeLong DM, Clarke-Pearson DL. Comparing the areas under two or more correlated receiver operating characteristic curves: a nonparametric approach. Biometrics. 1988 Sep;44(3):837-45.

Supplementary Figure 4:

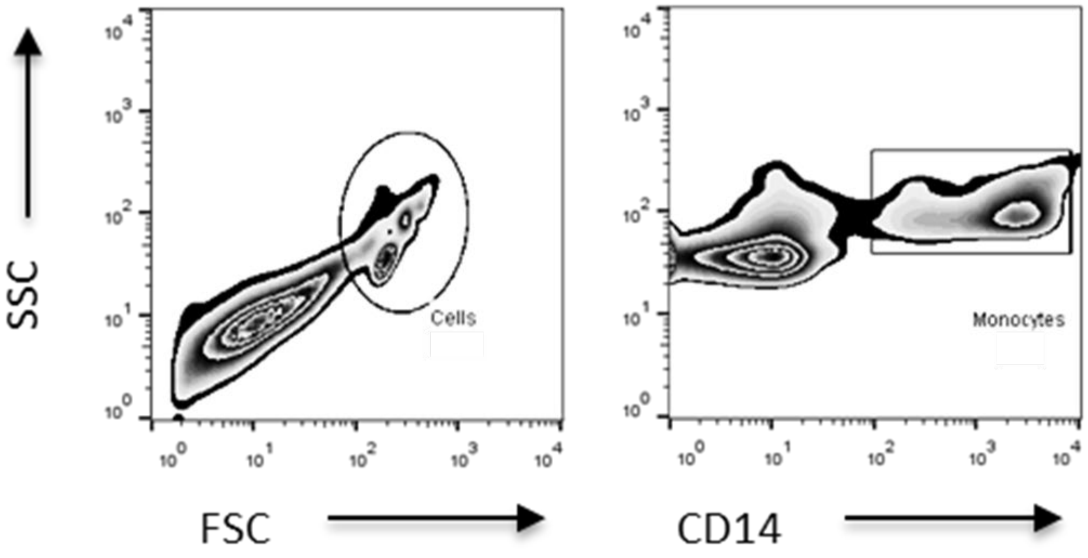

Suppl. figure 4. Monocyte gating strategy: For the positive selection, CD14 and complexity channel SSC dot plot were used. CD3 was used as a negative marker.

Supplementary Figure 5:

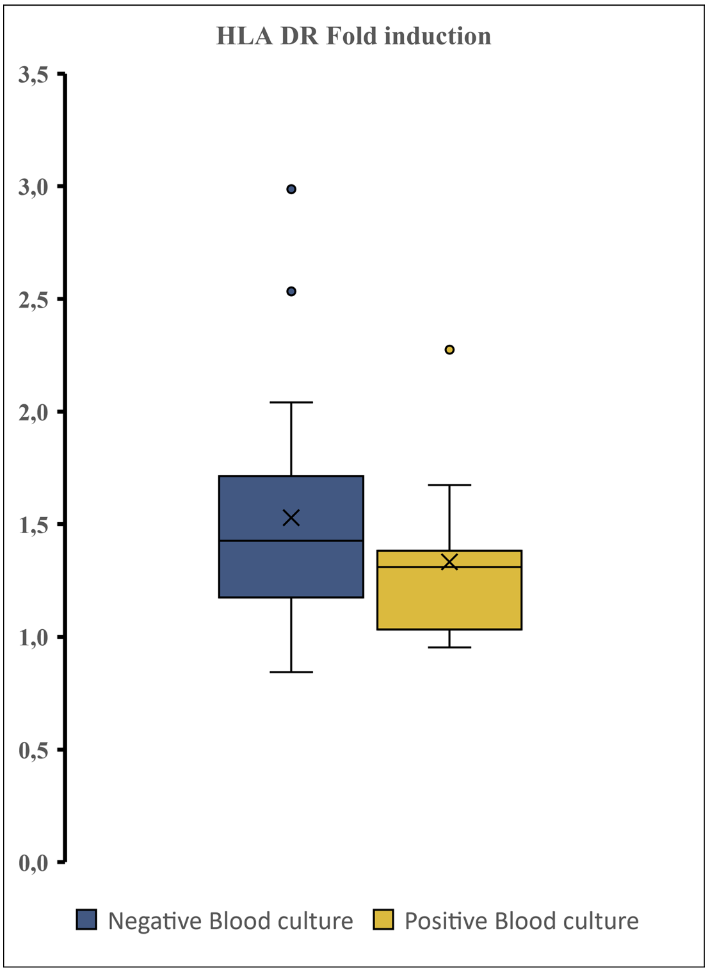

Suppl. figure 5. HLA-DR fold induction: 3h LPS challenge/basal (no stimulation). Box plots showing the medians, interquartile ranges (IQRs), minimums and maximums by group. Values over the maximum +1.5x IQR or below the minimum -1.5xIQR are considered outliers and are plotted outside the minimum and maximum range. X indicates the mean. HLA-DR values are cell-bound HLA-DR on monocytes expressed as mean intensity fluorescence (MIF) in arbitrary units (A. U.).

Supplementary Table 1: Clinical characteristics of reference cohort's patients

	Bacteremia negative (n=22)	Bacteremia positive (n=19)
**Age - Mean (SD)**	59.33 (24.15)	72.58 (16.1)
**Sex, female - n (%)**	14 (63.64)	9 (47,37)
**Diabetes Mellitus - n (%)**	4 (18.18)	6 (31.58)
**COPD - n (%)**	6 (27.27)	2 (10.53)
**Oncologic patient- n (%)**	3 (13.64)	1 (5.26)
**Cardiovascular disease - n (%)**	9 (40.91)	3 (15.79)
**Chronic Kidney Disease - n (%)**	2 (9.09)	3 (15.79)
**High Blood Pressure - n (%)**	13 (59.09)	12 (63.16)
**Smoker - n (%)**	4 (18.18)	1 (5.26)
**Suspected primary source:**		
**Urine - n (%)**	7 (31.82)	8 (42.11)
**Abdominal - n (%)**	7 (31.82)	3 (15.79)
**Respiratory - n (%)**	8 (36.36)	7 (36.84)
**Other - n (%)**	0	1 (5.26)

Supplementary Table 2: Patients in the validation cohort.

.

Age	Sex	Suspected primary source	Identified pathogen	Gram -	Gram +	TNFα (pg/ml)
49	Female	urine	E. coli	YES	NO	108.0
71	Male	abdominal	E. coli	YES	NO	155.0
78	Male	abdominal	E. coli	YES	NO	105.8
91	Female	abdominal	Klebsiella pneumoniae	YES	NO	85.0
88	Male	urine	E. coli	YES	NO	35.9
94	Female	urine	Proteus mirabilis	YES	NO	0.0
67	Female	abdominal	E. coli	YES	NO	0.0
32	Female	urine	E. coli	YES	NO	42.0
64	Male	abdominal	E. coli	YES	NO	22.4
93	Female	abdominal	E. coli	YES	NO	0.0
82	Male	urine	E. coli	YES	NO	0.0
86	Male	abdominal	E. coli	YES	NO	57.0
87	Female	abdominal	E. coli	YES	NO	330.3
83	Female	urine	E. coli	YES	NO	0.0
92	Male	urine	E. coli	YES	NO	66.1
70	Male	prostate	E. coli	YES	NO	0.0
48	Male	catheter	Citrobacter koseri	YES	NO	0.0
42	Female	abdominal	E. coli	YES	NO	0.0

83	Female	urine	E. coli	YES	NO	11.2
73	Male	abdominal	E. coli	YES	NO	56.0
93	Male	prostate	E. coli	YES	NO	87.3
70	Male	urine	Raoultella ornithinolytica	YES	NO	124.3
80	Male	abdominal	E. coli	YES	NO	117.7
57	Male	abdominal	E. coli	YES	NO	53.7
76	Male	prostate	E. coli	YES	NO	37.8
66	Female	respiratory	Haemophilus influenzae	YES	NO	0.0
88	Female	respiratory	Staphylococcus aureus	NO	YES	10.3
55	Male	catheter	Staphylococcus aureus	NO	YES	0.0
57	Male	catheter	Staphylococcus aureus	NO	YES	13.7
67	Female	catheter	Staphylococcus aureus	NO	YES	0.0
85	Male	left elbow	Staphylococcus aureus	NO	YES	11.3
87	Female	unknown	Staphylococcus aureus	NO	YES	9.8
83	Male	unknown	Enterococus Faecalis	NO	YES	9.3
78	Male	abdominal	Corynebacterium sp, E. Coli, staphylococcus sp. Enterococcus faecalis	YES	YES	137.1
84	Male	urine	Pseudomonas aeruginosa, others	YES	YES	207.1
